# Thymic self-antigen expression for immune tolerance and surveillance

**DOI:** 10.1186/s41232-022-00211-z

**Published:** 2022-09-03

**Authors:** Rayene Benlaribi, Qiao Gou, Hiroyuki Takaba

**Affiliations:** Department of Immunology, Graduate School of Medicine and Faculty of Medicine, The University of Tokyo, Tokyo, Japan

**Keywords:** Aire, Fezf2, Chd4, Immune tolerance, Cancer immunity, Regulatory T cells

## Abstract

T cells are a group of lymphocytes that play a central role in the immune system, notably, eliminating pathogens and attacking cancer while being tolerant of the self. Elucidating how immune tolerance is ensured has become a significant research issue for understanding the pathogenesis of autoimmune diseases as well as cancer immunity. T cell immune tolerance is established mainly in the thymic medulla by the removal of self-responsive T cells and the generation of regulatory T cells, this process depends mainly on the expression of a variety of tissue restricted antigens (TRAs) by medullary thymic epithelial cells (mTECs). The expression of TRAs is known to be regulated by at least two independent factors, Fezf2 and Aire, which play non-redundant and complementary roles by different mechanisms. In this review, we introduce the molecular logic of thymic self-antigen expression that underlies T cell selection for the prevention of autoimmunity and the establishment of immune surveillance.

## Introduction

Self/non-self discrimination is an inherent trait of functional T cells. They recognize their targets by binding to major histocompatibility complex (MHC) molecules and to short peptides loaded on them via their T cell receptor (TCR). The TCR is composed of two polypeptide chains (*α* and *β* chains) that are composed of a constant region and an antigen binding portion, namely the variable region. The variable region of *α* and *β* chains is constituted of V (variable) and J (joining) segments, or V (variable), D (diversity), and J (joining) segments, respectively [1]. To generate mature T cells, T cell progenitors originated in the bone marrow migrate to the thymic cortex where the TCR is generated by random somatic DNA rearrangement, known as the V (D) J recombination [2]. Each segment is represented by multiple copies in the genome and will be assembled by “cut and paste” DNA rearrangements where a pair of segments will be joined after cutting out the intervening DNA. One segment of each type is chosen from several, but sometimes many possibilities for assembly in a process that is the key behind antigen receptor diversity in mammal lymphocytes. Diversity is further amplified by adding or deleting small numbers of nucleotides at the junctions between the various segments [2]. This process explains the unlimited repertoire of potential antigen binding specificities encoded by a relatively small investment in germline capacity. But this comes at the cost of inevitably generating self-reactive T cells capable of causing peripheral tissue damage if not eliminated or controlled.

T cell tolerance is of particular importance, as it impacts B cell tolerance as well, through the need of T cell help in humoral responses [3]. Thus, failure of T cell tolerance results in many different autoimmune diseases. Mechanisms of T cell tolerance that occur in the thymus during their maturation are referred to as “central tolerance”. And additional tolerance mechanisms exist as complement, acting on mature circulating T cells and are referred to as “peripheral tolerance” [4].

In the thymic cortex, TCR expression on T cell precursors gives rise to CD4 and CD8 double-negative progenitors that will give rise to a large number of CD4 and CD8 double-positive (DP) thymocytes. The latter interact with cortical thymic epithelial cells (cTECs) that express self-antigens on MHC molecules. DP thymocytes expressing nonfunctional TCRs die by neglect, while those recognizing self-peptide–MHC complexes differentiate to CD4 or CD8 single-positive (SP) thymocytes in a process known as positive selection. CD8 and CD4 are co-receptor molecules that bind to non-polymorphic sites on MHC molecules restricting T cell specificity to MHC class I or MHC class II molecules, respectively [5]. SP thymocytes migrate to the thymic medulla, which serves a crucial function for T cell central tolerance induction, where they interact with self-peptide/MHC complexes expressed on the surface of antigen-presenting cells. SP thymocytes with high-affinity TCR for self-peptides represent a potential threat to health, therefore receive a lethal hit leading to their death by apoptosis in a process known as negative selection [4]. Apoptosis is not the only fate of autoreactive thymocytes, as a small fraction of CD4+ SP thymocytes is converted into CD25+ Foxp3+ regulatory T cells (Tregs), a cell type with suppressor activity that is required for the maintenance of immune homeostasis [6–9].

## Major antigen-presenting cells in the thymic medulla

Once the SP thymocytes are in the thymic medulla, those expressing self-reactive TCRs should be eliminated in a process called “negative selection.” To this end, SP thymocytes must interact with self-antigens presented on the cell surface of thymic antigen-presenting cells, in particular medullary thymic epithelial cells (mTECs), and dendritic cells (DCs).

***Medullary thymic epithelial cells***

mTECs produce a myriad of self-peptides known as tissue-restricted antigens (TRAs) representing, collectively, almost all peripheral transcripts, giving the medulla its crucial tolerogenic role [10]. For a long time, the consensus was that mTECs are divided to mTEC^lo^ and mTEC^hi^ [11], based on the low or high expression of MHC class II and CD80 and /or CD86 molecules, respectively. mTEC^hi^ express the autoimmune regulator (Aire) and are mature mTECs while mTEC^lo^ can be immature or post-Aire mTECs, with the understanding that immature mTEC^lo^ give rise to mature mTEC^hi^. The current view however, is that mTEC cell subset is much more heterogeneous with Podoplanin+ junctional TECs being the precursor of mature mTEC^hi^ rather than mTEC^lo^ expressing Ccl21, and Post-Aire mTECs being divided into two main subsets: Keratin-10+ mTECs and the recently identified thymic tuft cells [12–15]. Among these mTEC subsets, mTEC^hi^ expressing Aire and Fezf2 are considered as the professional TRA-producing mTECs.

***Dendritic cells***

The constitutive ablation of DCs results in spontaneous fatal autoimmunity [16], proving the importance of this cell subset in central tolerance induction. DCs do not have the ability to directly produce TRAs, they instead potentiate T cell selection process by presenting the antigens transferred to them from mTECs [17, 18], and presenting peripheral and blood-borne antigens [19–21]. Thymic DCs are divided into 70% conventional DCs (cDCs) and 30% plasmacytoid DCs (pDCs). cDCs are further divided into resident (Sirp *α*- CD8+) and migratory (Sirp *α*+ CD8-) cDCs, representing 70% and 30% of them, respectively [22]. pDCs are known to be poor antigen-presenting cells [23], and are thought not to contribute much to the thymic central tolerance. However, recent data challenged the accuracy of that idea. An in vitro study showed a role of thymic pDCs in Treg differentiation [24]. In vivo studies demonstrated the efficiency of pDCs in capturing peripheral soluble antigens and transporting them to the thymus [25, 26]. Migratory cDCs transport peripheral self-antigens into the thymus, and also capture and present blood-borne self-antigens. By contrast, resident cDCs capture and present self-antigens mainly found in the thymic microenvironment. Indeed, an in vitro study showed that resident Sirp *α*- CD8+ cDCs have the highest cross-presentation capacity compared to Sirp *α*+ CD8- migratory cDCs [27]. Recently, an additional subset of thymic DCs was described and named transendothelial DCs, owing to their localization in immediate proximity to thymic microvessels. This subset was shown to contribute to central tolerance by capturing blood-borne macromolecules and transporting them to the thymus [28].

## Regulators of the promiscuous expression of TRAs

***TRA expression regulated by Aire***

**Fig. 1:**
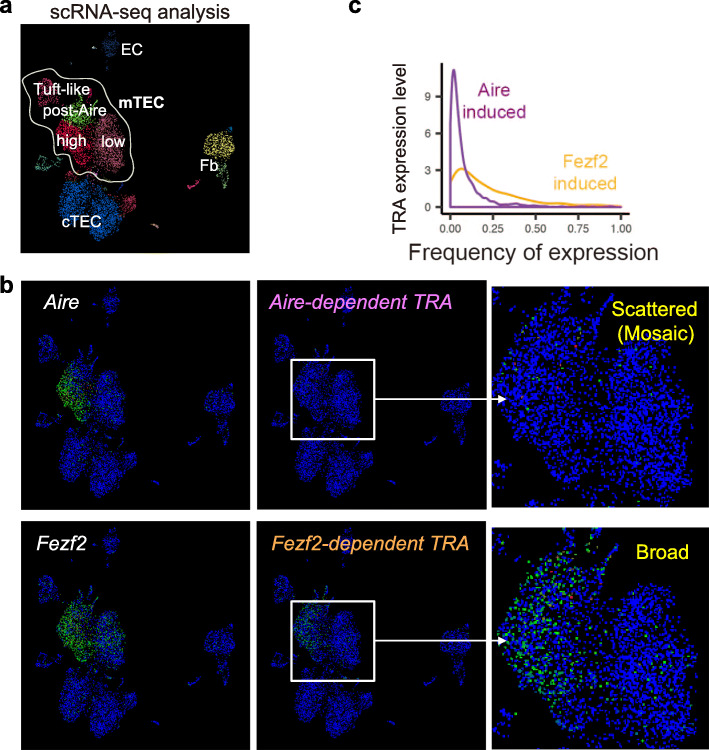
The expression pattern of TRAs in the mTECs by scRNA-seq analysis: **a** scRNA-seq analysis of thymic stromal cells. **b** Mosaic expression pattern of Aire-dependent TRA (top, Ins2). Broad expression of Fezf2-dependent TRAs (bottom, Muc1). **c** Fezf2-dependent TRAs are more highly expressed in a mature mTEC than Aire-dependent TRAs

The autoimmune regulator (Aire) gene was first described as the gene in which mutations caused APECED (autoimmune polyendocrinopathy-candidiasis-ectodermal dystrophy) [29, 30], a devastating rare genetic disorder that is characterized by a deregulated immune system. Autosomal recessive mutations in Aire gene cause the classical APECED, but some cases of APECED patients with Aire autosomal dominant mutations were noted [31, 32]. Mice lacking Aire gene showed multiorgan autoimmunity, importantly, these mice had reduced TRA expression in mTECs, revealing Aire’s major role in thymic central tolerance as a regulator of the ectopic expression of TRAs [33, 34]. During the past 25 years, extensive studies were conducted to reveal the complex mechanisms of Aire function [35], its binding partners [36], and the transcriptional programs regulating its own expression [37]. It is now understood that AIRE interacts with an abundance of proteins to exert its effect on a broad array of target genes. Aire contains SAND domain (Sp100, Aire, NucP41/P75, Deaf- 1), a DNA-binding domain. However, Aire’s SAND domain lacks the critical *α*-helical KDWK binding module, making it unable to recognize DNA target sites [38–40]. Aire working as a homomultimer was found to be recruited preferentially to inactive genes by interacting with proteins and histone markers involved in gene repression [41–47]. Thus, even though Aire regulates gene expression, it cannot be classified as a sequence-specific transcription factor. On the transcriptional starting sites of Aire-target genes, RNA polymerase II was found to be already engaged, but is stalled and unable to elongate the transcripts [48]. Aire then initiates a chain of events to release the stalled RNA polymerase II by recruiting the positive transcription elongation factor b (P-TEFb) via Bromodomain-containing protein 4 (BRD4). The transcriptional and epigenetic regulator BRD4 is able to release P-TEFb from its inhibitory complex [48–51]. Therefore, Aire stimulates the elongation of transcription. In addition, Aire interacts with DNA-PK, a member of the DNA damage machinery which is linked to transcriptional activation, but it’s still a debate whether DNA-PK activates Aire or it’s another way of how Aire is recruited to its target genes [40, 52–54]. Recently, localization studies found that Aire binds and activates super-enhancers, which are large clustered enhancer regions responsible for the transcriptional regulation of important cell-type-specific genes [55]. Aire drives TRA expression in a limited and scattered pattern (Fig. 1) and it was shown that co-expressed genes, present in only a subset of mTECs, clustered in the genome and showed enhanced chromatin accessibility [22, 56–59]. Of note, Aire-expressing mature mTECs (mTEC^hi^) experience radical changes in chromatin accessibility upon differentiation [60], which prepare the context for tissue-specific gene activation by Aire in mTECs. Indeed, a recent study showed that compromising the Brahma-associated factor (BAF) ATP-dependent chromatin remodeling complex changed the chromatin accessibility at different genome regions, resulting in a crucial reduction of the ectopic expression of Aire-dependent TRAs [60]. Aire functions expand beyond the regulation of TRA expression, it is involved in other processes in the thymus such as mTEC differentiation and antigen presentation, these functions are well reviewed by others [61, 62].

***TRA expression regulated by Fezf2***

Since the discovery of Aire, it has been reported that almost 60% of TRAs are expressed in an Aire-independent manner [10], indicating the existence of other regulators of TRA expression in mTEC. In 2015, our research team identified Fezf2 (Forebrain Embryonic Zinc Finger-Like Protein 2), a transcription factor which is expressed in human and mouse mTECs [63]. Although Aire is selectively expressed in mature mTEC^hi^, Fezf2 is mainly expressed in mTEChi, but also expressed in other subsets of mTECs. Microarray analysis of mTECs isolated from Fezf2 deficient mice revealed downregulation of Aire-independent TRA gene expression and mice deficient in Fezf2 specifically in mTECs developed autoimmune disease-like symptoms with autoantibody production and inflammatory cell infiltration into peripheral tissues. Interestingly, the target organs of autoimmunity that were observed in Aire-deficient and Fezf2-deficient mice were different, indicating that the distinct TRA expression in mTECs may be responsible for the difference in symptoms [63]. To identify genes regulated by Fezf2, RNA-seq analysis of mTECs from Fezf2-deficient mice compared to Aire-deficient and control mice was performed, and the data was integrated with tissue-specificity scores based on transcriptome data of various organs and cells obtained from a public database (BioGPS) [64]. The integrated analyses revealed differences in tissue specificity, for instance: Fezf2-regulated TRAs were highly expressed in the small intestine, but Aire-regulated TRAs were dominantly expressed in the eye. While, as explained above, genes regulated by Aire have inactive chromatin modifications and are expressed in a scattered pattern in the medulla, genes regulated by Fezf2 have active chromatin modifications and are expressed in a broad pattern in the medulla, in addition, Fezf2-induced genes are expressed at a higher level than Aire-induced genes [64] (Fig. 1). These observations indicate that the regulation of gene expression by Fezf2 and Aire is different at the epigenetic level.

**Fig. 2:**
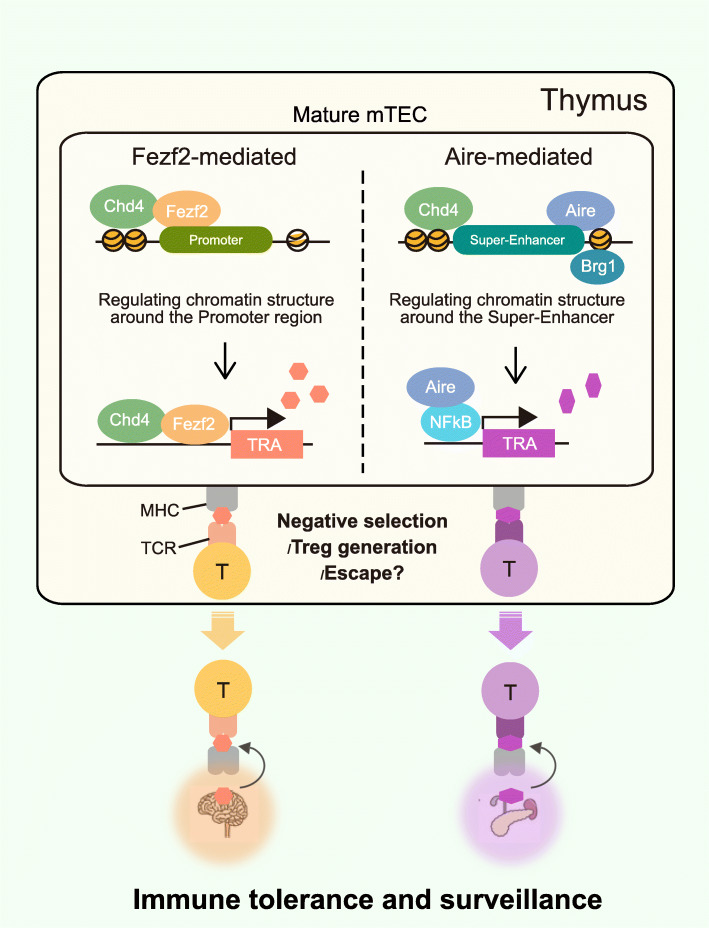
TRA expression mechanism by Fezf2 or Aire with Chd4 in mTECs in the thymus for immune homeostasis and surveillance: Super-enhancer mediated TRA expression by Aire (right), typical-enhancer mediated TRA expression by Fezf2 which forms NURD complex with Chd4 on the promoter regions (left). TRA-recognizing T cells differentiate into Treg cells or escape as autoreactive T cells from the thymus. Such T cells migrate to the secondary lymphoid organs and are distributed in specific peripheral tissues for immune homeostasis or surveillance

Although several epigenetic regulators that interact with Aire have been identified, not much is known about the recently discovered TRA expression-regulator Fezf2. Tomofuji et al. found the chromatin remodeling molecule Chd4 to interact with Fezf2 following comprehensive screening of Fezf2-interacting molecules by immunoprecipitation-mass spectrometry and western blotting. Chd4 is a significant component of the Nucleosome remodeling deacetylase (NuRD) complex, a vital complex involved in regulating chromatin structure, indicating that Fezf2, similar to Aire, acts on predefined chromatin landscape. It was found that Fezf2 binds to other components of the NuRD complex such as histone deacetylase HDAC1/2 indicating that Fezf2 forms a NuRD complex with Chd4 in mTECs and regulates gene expression [64]. RNA-seq analysis of Chd4-deficient mTECs revealed that genes regulated by Fezf2 and Chad4 overlap. ATAC-seq analysis showed that Fezf2 and Chd4 regulate chromatin accessibility in the same genomic regions and are involved in gene expression, especially in the promoter region. These results indicate that Fezf2 and Chd4 cooperatively regulate the chromatin structure near the promoter and typical enhancer regions and control the expression of a group of genes, including TRAs. Interestingly, while Aire and Chd4 do not interact with each other, RNA-seq analysis of Chd4-deficient mTECs revealed that Chd4 controls some of the genes regulated by Aire. ATAC-seq data demonstrated that both Chd4 and Aire are involved in regulating chromatin accessibility in the super-enhancer region as Chd4 preferentially regulates the chromatin accessibility of super-enhancers reportedly regulated by Aire [55]. In addition, genes whose expression was decreased by the inhibition of super-enhancer activity by I-BET 151 coincided with those whose expression was decreased by the loss of Chd4. Furthermore, using single-cell RNA-seq data, there was a co-expression cluster of genes near the super-enhancer and genes whose expression was regulated by Chd4 or Aire. However, Fezf2 does not induce TRA gene expression via super-enhancers (Fig. 2). These results suggest that Chd4 and Aire cooperatively regulate the chromatin structure of the super-enhancer region for specific TRA genes, even though they do not interact directly. In sum, Chd4 acts on two different transcriptional regulators, Fezf2 and Aire, and is involved in the regulation of the expression of different gene subsets. Of note, TEC-specific Chd4 deficient mice showed autoimmunity symptoms with infiltration of T cells into peripheral tissues and autoantibody production, reflecting the abnormal expression of TRAs in mTECs which impaired T cell selection. Along with the regulation of TRA expression, Fezf2, so far, was found to affect the number of mTECs and cytokine production, its implication in other roles needs to be further investigated [65].

## Antigens recognized by T cells

***Self-antigens in the development of Tregs***

Treg generation is defective in Aire and Fezf2 deficient mice, indicating that the thymic ectopic expression of self-antigens by Aire and Fezf2 is imperative for Treg development [63, 66, 67]. In the thymic medulla, while positive selection is driven by a low degree of TCR self-reactivity and negative selection by a strong one, a strength of reactivity in between will lead to clonal diversion of CD4+ SP thymocytes to Tregs [68]. Treg generation in the thymus is a two-step process, the first is TCR signaling-dependent, leading to the development of Treg precursors: CD25+ Foxp3- and CD25- Foxp3^lo^. The second step is triggered by cytokines, IL-2 or IL-15, that convert Treg precursors to CD25+ Foxp3+ mature Tregs [69–73]. Treg development from different precursors may indicate the heterogeneity of Tregs generated in the thymus. Mature Tregs then migrate to peripheral lymphoid and non-lymphoid tissues to fulfill their diverse roles [74, 75], and their distribution depends on the recognition of their cognate self-antigens. In addition, once Tregs are in a specific tissue, they adjust their transcriptomic profile [76]. More recently, single-cell RNA-seq revealed that Tregs are actually primed for tissue-specific migration in lymphoid organs [77–79]. Thus, Tregs are a heterogeneous subpopulation, and those with the same TCR specificity express tissue-specific genes and migrate to their respective tissue where they accumulate and adapt their transcriptional phenotype [80, 81]. This is in alliance with the fact that in the periphery, unlike conventional T cells, Tregs continuously receive TCR signals and inducible ablation of the TCR results in Treg cell dysfunction [82–84].

***Self-antigens in autoimmune diseases***

Several self-antigens have been identified to be targeted in autoimmune diseases in human patients and rodent models [85]. In many animal models of autoimmune diseases, immunization with the related autoantigen or transfer of autoreactive lymphocytes can induce the disease. For example, mouse immunization with myelin oligodendrocyte glycoprotein (MOG) is very efficient to induce the experimental autoimmune encephalomyelitis (EAE) with clinical and pathological similarities to multiple sclerosis (MS) [86, 87]. Peripheral blood lymphocytes from MS patients were reactive to MOG and had a proliferative response, suggesting an important role for cell reactivity against MOG in the pathogenesis of MS [87, 88]. Indeed, the transfer of MOG-specific CD4+ T cells to naïve mice can induce EAE [89]. Knowing that MOG is expressed in mouse and human mTECs [90, 91], several studies proved that MOG induces incomplete tolerance of CD4+T cells with concomitant Treg induction and that public anti-MOG T cell repertoires are selected for [92–94]. Meanwhile, loss of myelin proteolipid protein (Plp) or insulin 2 (Ins2) leads to the decrease of Plp- or Ins2-responsive Treg cells in the thymus, respectively [95, 96]. These studies suggest that autoreactive Tregs are agonistically selected for upon interactions with self-antigens. This mode of selection provides implications for the protective function of Treg cells in the periphery and for the design of antigen-specific immunotherapy. Thus, further studies to identify the cellular features (TRA peptide-TCR-T cell gene profile) of self-reactive T cells including Tregs may help to develop new immune treatments.

***Tumor-associated antigens***

For an effective anti-tumor response, the recognition of tumor antigens is imperative. Some antigens expressed abnormally by cancer cells, named tumor-associated antigens (TAAs), are self-antigens that can be presented on mTECs. Indeed, several antigens that are reported to be TAAs were found to be expressed on mTECs, such as Muc1 and CEA expressed in lung and breast cancer respectively [56] and gp100, which is a melanoma antigen, was found to be expressed in mTECs in an Aire-dependent manner [97]. The expression of TAAs on mTECs leads to the elimination of tumor-reactive T cells and the generation of tumor-recognizing Tregs. The expression of carcinoembryonic antigen (CEA) in mTECs impaired the anti-tumor CD4+ T cell repertoire [98]. On the other hand, a thymic-derived Treg population that is reactive to an Aire-dependent prostate-associated self-antigen has been reported to be enriched in the tumor microenvironment of oncogene-driven prostate cancer [99]. The elimination of tumor-reactive T cells and the generation of tumor-recognizing Tregs compromise the effectiveness of anti-tumor responses. Accordingly, mTECs from Aire-deficient mice show decreased expression of Trp1 (an Aire-dependent self-antigen expressed in melanoma cells) and an increased number of Trp1-responsive T cells, leading to an enhanced anti-tumor response [100]. Similarly, the blockade of RANK signaling, which is important for Aire expression, enhanced the survival in a melanoma mouse model [101]. Interestingly, Fezf2 suppresses the expression of some self-antigens among which are some TAAs such as Mesothelin (Msln), which is known to be overexpressed in several human cancers, implying the possibility of the existence of a regulatory mechanism that allows the generation of an antitumor immune defense [63, 64]. One of the breakthroughs in cancer therapy in recent years is immune checkpoint inhibitors that overcome the unresponsiveness of the immune system to tumor cells. However, there still are many patients who do not respond to checkpoint therapy, and one of the reasons for this is thought to be the immunosuppressive tumor Tregs. Tregs interfere with cancer immune surveillance by recognizing TAAs and suppressing antitumor immunity. Although a lot of studies focus on the mechanism of immunosuppressive molecules specifically expressed on Tregs, little is known about the origin of tumor Tregs, especially the identity of TAAs that induce Treg activity. In the future, it is necessary to understand the molecular mechanisms of the ontogeny of tumor antigen-responsive T cells in the thymus.

## Conclusions

In the thymic medulla, depending on the binding affinity and avidity of the TCR and self-antigens, differentiating T cells are eliminated by negative selection or converted into Tregs to ensure immune homeostasis. Some well-known self-antigens to be present in the thymic medulla are detected as the target in autoimmune diseases and cancers. This indicates that manipulating TRA presentation in the thymus could be a therapeutic strategy for autoimmune diseases and cancers. Two transcriptional regulators, Fezf2 and Aire, function in concert to drive the ectopic expression of TRAs in the human and mouse thymus, not only regulating the amount of TRA gene expression per mTEC cell, but also altering its expression pattern in the mTEC population as a whole. The expression levels and patterns of the TRA genes, possibly involved in the balance between Treg differentiation and autoreactive T cell leakage, need to be investigated in detail. Knowing that any taken individual has autoreactive T cells, the current consensus is that their presence in the periphery is a “defect in the system,” and Tregs’ major role is to cover up this loophole [102]. Based on some recent data demonstrating active processes limiting TRA expression in the thymus, this consensus might need to be revised. Fezf2, considering its role in the induction of TRA gene expression, can suppress the expression of some TRAs as well [63, 64]. In addition, Padonou et al. showed that in mTECs, Aire-dependent TRA genes escape the splicing-related factor Raver2, resulting in a low number of alternative splicing events, suggesting an incomplete Aire-dependent negative selection [103]. These observations could imply that the generation of autoreactive T cells is a physiological and beneficial process and might be explained by a role in immune surveillance and anti-tumor immunity. Hence, their control by Tregs in the periphery is just a regulating mechanism to avoid a time/space irrelevant activation of these autoreactive T cells. In summary, research on the mechanisms of autoantigen expression and T cell selection in the thymus is not only important for basic research, but is also expected to lead to the development of new immunological diagnostic and therapeutic techniques in the future.

## Data Availability

Not applicable.

## Abbreviations

MHC: Major histocompatibility complex molecules
TCR: T cell receptor
cTEC: Cortical thymic epithelial cell
TRA: Tissue-restricted antigen
Treg: Regulatory T cells
mTEC: Medullary thymic epithelial cell
DC: Dendritic cell
Fezf2: Forebrain embryonic zinc finger-like protein 2
Aire: Autoimmune regulator
NuRD: Nucleosome remodeling deacetylase
ATAC-seq: Assay for Transposase-Accessible Chromatin Sequencing
Mog: myelin oligodendrocyte glycoprotein
CNS: central nervous system
EAE: Experimental autoimmune encephalomyelitis
Plp: Myelin proteolipid protein
CEA: Carcinoembryonic antigen or carcinoembryonic antigen-related cell adhesion molecule 5
Muc1: Mucine 1
Ins2: Insulin 2
Trp1: Tyrosinase related protein 1
TAA: Tumor-associated antigen

## References

[1] Roth DB (2014). V(D)J Recombination: Mechanism, Errors, and Fidelity. Microbiol Spectr.

[2] Tonegawa S (1983). Somatic generation of antibody diversity. Nature.

[3] Murphy K, Weaver C (2017). Janeway’s Immunobiology, 9th ed (Chap. 10).

[4] Xing Y, Hogquist KA (2012). T-Cell Tolerance: Central and Peripheral. Cold Spring Harb Perspect Biol.

[5] Sprent J, Kishimoto H (2001). The thymus and central tolerance. Phil Trans R Soc B Biol Sci.

[6] Sakaguchi S, Yamaguchi T, Nomura T, Ono M (2008). Regulatory T Cells and Immune Tolerance. Cell.

[7] Lee HM, Bautista JL, Scott-Browne J, Mohan JF, Hsieh CS (2012). A Broad Range of Self-Reactivity Drives Thymic Regulatory T Cell Selection to Limit Responses to Self. Immunity.

[8] Ooi JD, Petersen J, Tan YH, Huynh M, Willett ZJ, Ramarathinam SH, Eggenhuizen PJ, Loh KL, Watson KA, Gan PY, Alikhan MA, Dudek NL, Handel A, Hudson BG, Fugger L, Power DA, Holt SG, Coates PT, Gregersen JW, Purcell AW, Holdsworth SR, La Gruta NL, Reid HH, Rossjohn J, Kitching AR (2017). Dominant protection from HLA-linked autoimmunity by antigen-specific regulatory T cells. Nature.

[9] Leonard JD, Gilmore DC, Dileepan T, Nawrocka WI, Chao JL, Schoenbach MH, Jenkins MK, Adams EJ, Savage PA (2017). Identification of Natural Regulatory T Cell Epitopes Reveals Convergence on a Dominant Autoantigen. Immunity.

[10] Derbinski J, Gäbler J, Brors B, Tierling S, Jonnakuty S, Hergenhahn M, Peltonen L, Walter J, Kyewski B (2005). Promiscuous gene expression in thymic epithelial cells is regulated at multiple levels. J Exp Med.

[11] Gray DH, Seach N, Ueno T, Milton MK, Liston A, Lew AM, Goodnow CC, Boyd RL (2006). Developmental kinetics, turnover, and stimulatory capacity of thymic epithelial cells. Blood.

[12] Lkhagvasuren E, Sakata M, Ohigashi I, Takahama Y (2013). Lymphotoxin *β* Receptor Regulates the Development of CCL21-Expressing Subset of Postnatal Medullary Thymic Epithelial Cells. J Immunol.

[13] Onder L, Nindl V, Scandella E, Chai Q, Cheng HW, Caviezel-Firner S, Novkovic M, Bomze D, Maier R, Mair F, Ledermann B, Becher B, Waisman A, Ludewig B (2015). Alternative NF- *κ*B signaling regulates mTEC differentiation from podoplanin-expressing presursors in the cortico-medullary junction. Eur J Immunol.

[14] Miller CN, Proekt I, von Moltke J, Wells KL, Rajpurkar AR, Wang H, Rattay K, Khan IS, Metzger TC, Pollack JL (2018). Thymic tuft cells promote an il-4-enriched medulla and shape thymocyte development. Nature.

[15] Bornstein C, Nevo S, Giladi A, Kadouri N, Pouzolles M, Gerbe F, David E, Machado A, Chuprin A, Tóth B (2018). Single-cell mapping of the thymic stroma identifies il-25-producing tuft epithelial cells. Nature.

[16] Ohnmacht C, Pullner A, King SBS, Drexler I, Meier S, Brocker T, Voehringer D (2009). Constitutive ablation of dendritic cells breaks self-tolerance of CD4 T cells and results in spontaneous fatal autoimmunity. J Exp Med.

[17] Gallegos AM, Bevan MJ (2004). Central Tolerance to Tissue-specific Antigens Mediated by Direct and Indirect Antigen Presentation. J Exp Med.

[18] Koble C, Kyewski B (2009). The thymic medulla: a unique microenvironment for intercellular self-antigen transfer. J Exp Med.

[19] Baba T, Nakamoto Y, Mukaida N (2009). Crucial Contribution of Thymic Sirp *α* + Conventional Dendritic Cells to Central Tolerance against Blood-Borne Antigens in a CCR2-Dependent Manner. J Immunol.

[20] Atibalentja DF, Byersdorfer CA, Unanue ER (2009). Thymus-Blood Protein Interactions Are Highly Effective in Negative Selection and Regulatory T Cell Induction. J Immunol.

[21] Atibalentja DF, Murphy KM, Unanue ER (2011). Functional Redundancy between Thymic CD8 *α* + and Sirp *α* + Conventional Dendritic Cells in Presentation of Blood-Derived Lysozyme by MHC Class II Proteins. J Immunol.

[22] Klein L, Kyewski B, Allen PM, Hogquist KA (2014). Positive and negative selection of the T cell repertoire: What thymocytes see (and don’t see). Nat Rev Immunol.

[23] Villadangos JA, Young L (2008). Antigen-presentation properties of plasmacytoid dendritic cells. Immunity.

[24] Wirnsberger G, Mair F, Klein L (2009). Regulatory T cell differentiation of thymocytes does not require a dedicated antigen-presenting cell but is under T cell-intrinsic developmental control. Proc Natl Acad Sci U S A.

[25] Li J, Park J, Foss D, Goldschneider I (2009). Thymus-homing peripheral dendritic cells constitute two of the three major subsets of dendritic cells in the steady-state thymus. J Exp Med.

[26] Hadeiba H, Lahl K, Edalati A, Oderup C, Habtezion A, Pachynski R, Nguyen L, Ghodsi A, Adler S, Butcher EC (2012). Plasmacytoid Dendritic Cells Transport Peripheral Antigens to the Thymus to Promote Central Tolerance. Immunity.

[27] Proietto AI, Lahoud MH, Wu L (2008). Distinct functional capacities of mouse thymic and splenic dendritic cell populations. Immunol Cell Biol.

[28] Vollmann EH, Rattay K, Barreiro O, Thiriot A, Fuhlbrigge RA, Vrbanac V, Kim KW, Jung S, Tager AM, von Andrian UH (2021). Specialized transendothelial dendritic cells mediate thymic T-cell selection against blood-borne macromolecules. Nat Commun.

[29] Nagamine K, Peterson P, Scott HS, Kudoh J, Minoshima S, Heino M, Krohn KJE, Lalioti MD, Mullis PE, Antonarakis SE, Kawasaki K, Asakawa S, Ito F, Shimizu N (1997). Positional cloning of the APECED gene. Nat Genet.

[30] Aaltonen J, Björses P, Perheentupa J, Horelli–Kuitunen N, Palotie A, Peltonen L, Lee YS, Francis F, Henning S, Thiel C, Leharach H, Yaspo M (1997). An autoimmune disease, APECED, caused by mutations in a novel gene featuring two PHD-type zinc-finger domains. Nat Genet.

[31] Cetani F, Barbesino G, Borsari S, Pardi E, Cianferotti L, Pinchera A, Marcocci C (2001). A Novel Mutation of the Autoimmune Regulator Gene in an Italian Kindred with Autoimmune Polyendocrinopathy-Candidiasis-Ectodermal Dystrophy, Acting in a Dominant Fashion and Strongly Cosegregating with Hypothyroid Autoimmune Thyroiditis. J Clin Endocrinol Metab.

[32] Bruserud Ø, Oftedal BE, Wolff AB, Husebye ES (2016). Aire-mutations and autoimmune disease. Curr Opin Immunol.

[33] Ramsey C, Winqvist O, Puhakka L, Halonen M, Moro A, Kämpe O, Eskelin P, Pelto-Huikko M, Peltonen L (2002). Aire deficient mice develop multiple features of APECED phenotype and show altered immune response. Hum Mol Genet.

[34] Anderson MS, Venanzi ES, Klein L, Chen Z, Berzins SP, Turley SJ, von Boehmer H, Bronson R, Dierich A, Benoist C, Mathis D (2002). Projection of an immunological self shadow within the thymus by the aire protein. Science (New York, N.Y.).

[35] Passos GA, Speck-Hernandez CA, Assis AF, Mendes-da-Cruz DA (2018). Update on Aire and thymic negative selection. Immunology.

[36] Abramson J, Goldfarb Y (2016). AIRE: From promiscuous molecular partnerships to promiscuous gene expression. Eur J Immunol.

[37] Herzig Y, Nevo S, Bornstein C, Brezis MR, Ben-Hur S, Shkedy A, Eisenberg-Bord M, Levi B, Delacher M, Goldfarb Y, David E, Weinberger L, Viukov S, Ben-Dor S, Giraud M, Hanna JH, Breiling A, Lyko F, Amit I, Feuerer M, Abramson J (2017). Transcriptional programs that control expression of the autoimmune regulator gene Aire. Nat Immunol.

[38] Bottomley MJ, Collard MW, Huggenvik JI, Liu Z, Gibson TJ, Sattler M (2001). The SAND domain structure defines a novel DNA-binding fold in transcriptional regulation. Nat Struct Biol.

[39] Koh AS, Kuo AJ, Sang YP, Cheung P, Abramson J, Bua D, Carney D, Shoelson SE, Gozani O, Kingston RE, Benoist C, Mathis D (2008). Aire employs a histone-binding module to mediate immunological tolerance, linking chromatin regulation with organ-specific autoimmunity. Proc Natl Acad Sci U S A.

[40] žumer K, Low AK, Jiang H, Saksela K, Peterlin BM (2012). Unmodified Histone H3K4 and DNA-Dependent Protein Kinase Recruit Autoimmune Regulator to Target Genes. Mol Cell Biol.

[41] Halonen M, Kangas H, Rüppell T, Ilmarinen T, Ollila J, Kolmer M, Vihinen M, Palvimo J, Saarela J, Ulmanen I, Eskelin P (2004). APECED-causing mutations in AIRE reveal the functional domains of the protein. Hum Mutat.

[42] Ferguson BJ, Alexander C, Rossi SW, Liiv I, Rebane A, Worth CL, Wong J, Laan M, Peterson P, Jenkinson EJ, Anderson G, Scott HS, Cooke A, Rich T (2008). AIRE’s CARD Revealed, a New Structure for Central Tolerance Provokes Transcriptional Plasticity. J Biol Chem.

[43] Org T, Chignola F, Hetényi C, Gaetani M, Rebane A, Liiv I, Maran U, Mollica L, Bottomley MJ, Musco G, Peterson P (2008). The autoimmune regulator PHD finger binds to non-methylated histone H3K4 to activate gene expression. EMBO Rep.

[44] Org T, Rebane A, Kisand K, Laan M, Haljasorg U, Andreson R, Peterson P (2009). AIRE activated tissue specific genes have histone modifications associated with inactive chromatin. Hum Mol Genet.

[45] Waterfield M, Khan IS, Cortez JT, Fan U, Metzger T, Greer A, Fasano K, Martinez-Llordella M, Pollack JL, Erle DJ, Su M, Anderson MS (2014). The transcriptional regulator Aire coopts the repressive ATF7ip-MBD1 complex for the induction of immunotolerance. Nat Immunol.

[46] Sansom SN, Shikama-Dorn N, Zhanybekova S, Nusspaumer G, Macaulay IC, Deadman ME, Heger A, Ponting CP, Holländer GA (2014). Population and single-cell genomics reveal the Aire dependency, relief from Polycomb silencing, and distribution of self-antigen expression in thymic epithelia. Genome Res.

[47] Huoh Y-S, Wu B, Park S, Yang D, Bansal K, Greenwald E, Wong WP, Mathis D, Hur S (2020). Dual functions of Aire CARD multimerization in the transcriptional regulation of T cell tolerance. Nat Commun.

[48] Oven I, Brdičková N, Kohoutek J, Vaupotič T, Narat M, Peterlin BM (2007). AIRE Recruits P-TEFb for Transcriptional Elongation of Target Genes in Medullary Thymic Epithelial Cells. Mol Cell Biol.

[49] Giraud M, Yoshid H, Abramson J, Rahl PB, Young RA, Mathis D, Benoist C (2012). Aire unleashes stalled RNA polymerase to induce ectopic gene expression in thymic epithelial cells. Proc Natl Acad Sci U S A.

[50] Giraud M, Jmari N, Du L, Carallis F, Nieland TJF, Perez-Campo FM, Bensaude O, Root DE, Hacohen N, Mathis D, Benoist C (2014). An RNAi screen for Aire cofactors reveals a role for Hnrnpl in polymerase release and Aire-activated ectopic transcription. Proc Natl Acad Sci U S A.

[51] Yoshida H, Bansal K, Schaefer U, Chapman T, Rioja I, Proekt I, Anderson MS, Prinjha RK, Tarakhovsky A, Benoist C, Mathis D (2015). Brd4 bridges the transcriptional regulators, Aire and P-TEFb, to promote elongation of peripheral-tissue antigen transcripts in thymic stromal cells. Proc Natl Acad Sci U S A.

[52] Liiv I, Rebane A, Org T, Saare M, Maslovskaja J, Kisand K, Juronen E, Valmu L, Bottomley MJ, Kalkkinen N, Peterson P (2008). DNA-PK contributes to the phosphorylation of AIRE: Importance in transcriptional activity. Biochim Biophys Acta Mol Cell Res.

[53] žumer K, Saksela K, Peterlin BM (2013). The Mechanism of Tissue-Restricted Antigen Gene Expression by AIRE. J Immunol.

[54] Abramson J, Giraud M, Benoist C, Mathis D (2010). Aire’s Partners in the Molecular Control of Immunological Tolerance. Cell.

[55] Bansal K, Yoshida H, Benoist C, Mathis D (2017). The transcriptional regulator Aire binds to and activates super-enhancers. Nat Immunol.

[56] Pinto S, Michel C, Schmidt-Glenewinkel H, Harder N, Rohr K, Wild S, Brors B, Kyewski B (2013). Overlapping gene coexpression patterns in human medullary thymic epithelial cells generate self-antigen diversity. Proc Natl Acad Sci U S A.

[57] Sansom SN, Shikama-Dorn N, Zhanybekova S, Nusspaumer G, Macaulay IC, Deadman ME, Heger A, Ponting CP, Holländer GA (2014). Population and single-cell genomics reveal the Aire dependency, relief from Polycomb silencing, and distribution of self-antigen expression in thymic epithelia. Genome Res.

[58] Meredith M, Zemmour D, Mathis D, Benoist C (2015). Aire controls gene expression in the thymic epithelium with ordered stochasticity. Nat Immunol.

[59] Brennecke P, Reyes A, Pinto S, Rattay K, Nguyen M, Küchler R, Huber W, Kyewski B, Steinmetz LM (2015). Single-cell transcriptome analysis reveals coordinated ectopic gene-expression patterns in medullary thymic epithelial cells. Nat Immunol.

[60] Koh AS, Miller EL, Buenrostro JD, Moskowitz DM, Wang J, Greenleaf WJ, Chang HY, Crabtree GR (2018). Rapid chromatin repression by Aire provides precise control of immune tolerance article. Nat Immunol.

[61] Anderson MS, Su MA (2016). AIRE expands: New roles in immune tolerance and beyond. Nat Rev Immunol.

[62] Perniola R. Twenty Years of AIRE. Front Immunol. 2018;9(FEB). 10.3389/fimmu.2018.00098. 10.3389/fimmu.2018.00098 PMC5816566 29483906

[63] Takaba H, Morishita Y, Tomofuji Y, Danks L, Nitta T, Komatsu N, Kodama T, Takayanagi H (2015). Fezf2 Orchestrates a Thymic Program of Self-Antigen Expression for Immune Tolerance. Cell.

[64] Tomofuji Y, Takaba H, Suzuki HI, Benlaribi R, Martinez CDP, Abe Y, Morishita Y, Okamura T, Taguchi A, Kodama T, Takayanagi H (2020). Chd4 choreographs self-antigen expression for central immune tolerance. Nat Immunol.

[65] Takaba H, Takayanagi H (2017). The Mechanisms of T Cell Selection in the Thymus. Trends Immunol.

[66] Yang S, Fujikado N, Kolodin D, Benoist C, Mathis D (2015). Regulatory T cells generated early in life play a distinct role in maintaining self-tolerance. Science.

[67] Malchow S, Leventhal DS, Lee V, Nishi S, Socci ND, Savage PA (2016). Aire Enforces Immune Tolerance by Directing Autoreactive T Cells into the Regulatory T Cell Lineage. Immunity.

[68] Hsieh CS, Lee HM, Lio CWJ (2012). Selection of regulatory T cells in the thymus. Nat Rev Immunol.

[69] Lio CWJ, Hsieh CS (2008). A Two-Step Process for Thymic Regulatory T Cell Development. Immunity.

[70] Burchill MA, Yang J, Vang KB, Moon JJ, Chu HH, Lio CWJ, Vegoe AL, Hsieh CS, Jenkins MK, Farrar MA (2008). Linked T Cell Receptor and Cytokine Signaling Govern the Development of the Regulatory T Cell Repertoire. Immunity.

[71] Tai X, Erman B, Alag A, Mu J, Kimura M, Katz G, Guinter T, McCaughtry T, Etzensperger R, Feigenbaum L, Singer DS, Singer A (2013). Foxp3 Transcription Factor Is Proapoptotic and Lethal to Developing Regulatory T Cells unless Counterbalanced by Cytokine Survival Signals. Immunity.

[72] Mahmud SA, Manlove LS, Schmitz HM, Xing Y, Wang Y, Owen DL, Schenkel JM, Boomer JS, Green JM, Yagita H, Chi H, Hogquist KA, Farrar MA (2014). Costimulation via the tumor-necrosis factor receptor superfamily couples TCR signal strength to the thymic differentiation of regulatory T cells. Nat Immunol.

[73] Owen DL, Sjaastad LE, Farrar MA (2019). Regulatory T Cell Development in the Thymus. J Immunol.

[74] Wei S, Kryczek I, Zou W (2006). Regulatory T-cell compartmentalization and trafficking. Blood.

[75] Muñoz-Rojas AR, Mathis D (2021). Tissue regulatory T cells: regulatory chameleons. Nat Rev Immunol.

[76] Zemmour D, Zilionis R, Kiner E, Klein AM, Mathis D, Benoist C (2018). Single-cell gene expression reveals a landscape of regulatory T cell phenotypes shaped by the TCR article. Nat Immunol.

[77] Li C, DiSpirito JR, Zemmour D, Spallanzani RG, Kuswanto W, Benoist C, Mathis D (2018). TCR Transgenic Mice Reveal Stepwise, Multi-site Acquisition of the Distinctive Fat-Treg Phenotype. Cell.

[78] Miragaia RJ, Gomes T, Chomka A, Jardine L, Riedel A, Hegazy AN, Whibley N, Tucci A, Chen X, Lindeman I, Emerton G, Krausgruber T, Shields J, Haniffa M, Powrie F, Teichmann SA (2019). Single-Cell Transcriptomics of Regulatory T Cells Reveals Trajectories of Tissue Adaptation. Immunity.

[79] Delacher M, Imbusch CD, Hotz-Wagenblatt A, Mallm JP, Bauer K, Simon M, Riegel D, Rendeiro AF, Bittner S, Sanderink L, Pant A, Schmidleithner L, Braband KL, Echtenachter B, Fischer A, Giunchiglia V, Hoffmann P, Edinger M, Bock C, Rehli M, Brors B, Schmidl C, Feuerer M (2020). Precursors for Nonlymphoid-Tissue Treg Cells Reside in Secondary Lymphoid Organs and Are Programmed by the Transcription Factor BATF. Immunity.

[80] Pohar J, Simon Q, Fillatreau S (2018). Antigen-Specificity in the Thymic Development and Peripheral Activity of CD4+FOXP3+ T Regulatory Cells. Front Immunol.

[81] Shevyrev D, Tereshchenko V (2020). Treg Heterogeneity, Function, and Homeostasis. Front Immunol.

[82] Moran AE, Holzapfel KL, Xing Y, Cunningham NR, Maltzman JS, Punt J, Hogquist KA (2011). T cell receptor signal strength in Treg and iNKT cell development demonstrated by a novel fluorescent reporter mouse. J Exp Med.

[83] Vahl JC, Drees C, Heger K, Heink S, Fischer JC, Nedjic J, Ohkura N, Morikawa H, Poeck H, Schallenberg S, Rieß D, Hein MY, Buch T, Polic B, Schönle A, Zeiser R, Schmitt-Gräff A, Kretschmer K, Klein L, Korn T, Sakaguchi S, Schmidt-Supprian M (2014). Continuous T Cell Receptor Signals Maintain a Functional Regulatory T Cell Pool. Immunity.

[84] Levine AG, Arvey A, Jin W, Rudensky AY (2014). Continuous requirement for the TCR in regulatory T cell function. Nat Immunol.

[85] Mocci S, Lafferty K, Howard M (2000). The role of autoantigens in autoimmune disease. Curr Opin Immunol.

[86] Linnington C, Webb M, Woodhams PL (1984). A novel myelin-associated glycoprotein defined by a mouse monoclonal antibody. J Neuroimmunol.

[87] Bernard CCA, Johns TG, Slavin A, Ichikawa M, Ewing C, Liu J, Bettadapura J (1997). Myelin oligodendrocyte glycoprotein: A novel candidate autoantigen in multiple sclerosis. J Mol Med.

[88] De Rosbo NK, Milo R, Lees MB, Burger D, Bernard CCA, Ben-Nun A (1993). Reactivity to myelin antigens in multiple sclerosis. Peripheral blood lymphocytes respond predominantly to myelin oligodendrocyte glycoprotein. J Clin Investig.

[89] Mendel I, de Rosbo NK, Ben-Nun A (1995). A myelin oligodendrocyte glycoprotein peptide induces typical chronic experimental autoimmune encephalomyelitis in H-2b mice: Fine specificity and T cell receptor V *β* expression of encephalitogenic T cells. Eur J Immunol.

[90] Derbinski J, Schulte A, Kyewski B, Klein L (2001). Promiscuous gene expression in medullary thymic epithelial cells mirrors the peripheral self. Nat Immunol.

[91] Gotter J, Brors B, Hergenhahn M, Kyewski B (2004). Medullary Epithelial Cells of the Human Thymus Express a Highly Diverse Selection of Tissue-specific Genes Colocalized in Chromosomal Clusters. J Exp Med.

[92] Delarasse C, Daubas P, Mars LT, Vizler C, Litzenburger T, Iglesias A, Bauer J, Della Gaspera B, Schubart A, Decker L, Dimitri D, Roussel G, Dierich A, Amor S, Dautigny A, Liblau R, Pham-Dinh D (2003). Myelin/oligodendrocyte glycoprotein–deficient (MOG-deficient) mice reveal lack of immune tolerance to MOG in wild-type mice. J Clin Investig.

[93] Fazilleau N, Delarasse C, Sweenie CH, Anderton SM, Fillatreau S, Lemonnier FA, Pham-Dinh D, Kanellopoulos JM (2006). Persistence of autoreactive myelin oligodendrocyte glycoprotein (MOG)-specific T cell repertoires in MOG-expressing mice. Eur J Immunol.

[94] Lucca LE, Axisa P, Aloulou M, Perals C, Ramadan A, Rufas P, Kyewski B, Derbinski J, Fazilleau N, Mars LT, Liblau RS (2016). Myelin oligodendrocyte glycoprotein induces incomplete tolerance of CD4 + T cells specific for both a myelin and a neuronal self-antigen in mice. Eur J Immunol.

[95] Lee T, Sprouse ML, Banerjee P, Bettini M, Bettini ML (2017). Ectopic Expression of Self-Antigen Drives Regulatory T Cell Development and Not Deletion of Autoimmune T Cells. J Immunol.

[96] Hassler T, Urmann E, Teschner S, Federle C, Dileepan T, Schober K, Jenkins MK, Busch DH, Hinterberger M, Klein L (2019). Inventories of naive and tolerant mouse CD4 T cell repertoires reveal a hierarchy of deleted and diverted T cell receptors. Proc Natl Acad Sci U S A.

[97] Träger U, Sierro S, Djordjevic G, Bouzo B, Khandwala S, Meloni A, Mortensen M, Simon AK. The immune response to melanoma is limited by thymic selection of self-antigens. PLoS ONE. 2012;7(4). 10.1371/journal.pone.0035005. 10.1371/journal.pone.0035005 PMC3323626 22506061

[98] Bos R, Van Duikeren S, Van Hall T, Kaaijk P, Taubert R, Kyewski B, Klein L, Melief CJM, Offringa R (2005). Expression of a natural tumor antigen by thymic epithelial cells impairs the tumor-protective CD4+ T-cell repertoire. Cancer Res.

[99] Malchow S, Leventhal DS, Nishi S, Fischer BI, Shen L, Paner GP, Amit AS, Kang C, Geddes JE, Allison JP, Socci ND, Savage PA (2013). Aire-Dependent Thymic Development of Tumor-Associated Regulatory T Cells. Science.

[100] Zhu M-L, Nagavalli A, Su MA (2013). Aire Deficiency Promotes TRP-1–Specific Immune Rejection of Melanoma. Cancer Res.

[101] Khan IS, Mouchess ML, Zhu ML, Conley B, Fasano KJ, Hou Y, Fong L, Su MA, Anderson MS (2014). Enhancement of an anti-tumor immune response by transient blockade of central T cell tolerance. J Exp Med.

[102] Danke NA, Koelle DM, Yee C, Beheray S, Kwok WW (2004). Autoreactive T Cells in Healthy Individuals. J Immunol.

[103] Padonou F, Gonzalez V, Provin N, Yayilkan S, Jmari N, Maslovskaja J, Kisand K, Peterson P, Irla M, Giraud M. Aire-dependent transcripts escape Raver2-induced splice-event inclusion in the thymic epithelium. EMBO Rep. 2022;1–16. 10.15252/embr.202153576. 10.15252/embr.202153576 PMC8892270 35037357

